# Absence of Pericarditis Recurrence in Rilonacept-Treated Patients With COVID-19 and SARS-CoV-2 Vaccination: Results From the RHAPSODY Long-term Extension

**DOI:** 10.1016/j.cjco.2024.02.002

**Published:** 2024-03-04

**Authors:** Antonio Brucato, Lucia Trotta, Michael Arad, Paul C. Cremer, Antonella Insalaco, Marc Klutstein, Martin LeWinter, David Lin, Sushil A. Luis, Yishay Wasserstrum, JoAnn Clair, Sheldon Wang, Allan L. Klein, Massimo Imazio, John F. Paolini, RHAPSODY Investigators, Antonio Abbate, Wael Abo-Auda, Asif Akhtar, Michael Arad, Shaul Atar, Bipul Baibhav, Antonio Brucato, Sean Collins, David Colquhoun, Paul Cremer, David Cross, Girish Dwivedi, Alon Eisen, Nahum Freedberg, Shmuel Fuchs, Eliyazar Gaddam, Marco Gattorno, Eli Gelfand, Paul Grena, Majdi Halabi, David Harris, Massimo Imazio, Antonella Insalaco, Amin Karim, Allan Klein, Kirk Knowlton, Apostolos Kontzias, Robert Kornberg, Faisal Latif, David Leibowitz, Martin LeWinter, Pey Wen Lou, David Lin, S. Allen Luis, Stephen Nicholls, John Petersen, Michael Portman, Philip Roberts-Thomson, Elad Schiff, Robert Siegel, Michael Stokes, Paul Sutej, Samuel Wittekind, Valentin Witzling, Robert Zukermann

**Affiliations:** o University of Virginia, Charlottesville, Virginia, USA; p CardioVoyage, McKinney, Texas, USA; q BI Research Center, Houston, Texas, USA; r Chaim Sheba Medical Center, Ramat Gan, Israel; s Galilee Medical Center, Nahariya, Israel; t Rochester General Hospital, Rochester, New York, USA; u ASST Fatebenefratelli Sacco - Ospedale Fatebenefratelli e Oftalmico, Milan, Italy; v Vanderbilt University Medical Center, Nashville, Tennessee, USA; w Core Research Group, Milton, Queensland, Australia; X Cleveland Clinic, Cleveland, Ohio, USA; y HeartCare Partners Clinical Research Unit, Milton, Queensland, Australia; z Fiona Stanley Hospital, Murdoch, Western Australia, Australia; aa Rabin Medical Center, Petach Tikva, Israel; bb HaEmek Medical Center, Afula, Israel; cc Assaf Harofe Medical Center, Tzrifin, Israel; dd Loretto Hospital, Chicago, Illinois, USA; ee Istituto G Gaslini Ospedale Pediatrico IRCCS, Genova, Italy; ff Beth Israel Deaconess Medical Center, Boston, Massachusetts, USA; gg Cardiology Consultants of Philadelphia, Yardley, Pennsylvania, USA; hh Ziv Medical Center, Zefat, Israel; ii University of Cincinnati, Cincinnati, Ohio, USA; jj Azienda Ospedaliero Città della Salute e della Scienza di Torino, Turin, Italy; kk Ospedale Pediatrico Bambino Gesù, Rome, Italy; ll Angiocardiac Care of Texas PA, Houston, Texas, USA; mm Cleveland Clinic, Cleveland, Ohio, USA; nn Intermountain Healthcare, Murray, Utah, USA; oo Stony Brook University School of Medicine Stony Brook, New York, USA; pp Icahn School of Medicine at Mount Sinai New York, New York, USA; qq Oklahoma City VA Medical Center – National Association of Veterans’ Research and Education Foundations (NAVREF), Oklahoma City, Oklahoma, USA; uu Hadassah University Hospital Mount Scopus Jerusalem, Israel; vv University of Vermont Medical Center, Burlington, Vermont, USA; ww Minneapolis Heart Institute Foundation Minneapolis, Minnesota, USA; xx GenesisCare – Cardiology Research, Doncaster, East Victoria, Australia; yy Mayo Clinic, Rochester, Minnesota, USA; zz Monash Health, Monash Medical Centre Clayton, Victoria, Australia; aaa Swedish Medical Center Seattle, Washington, USA; bbb Seattle Children’s Hospital Seattle, Washington, USA; ccc Royal Hobart Hospital, Hobart, Tasmania, Australia; ddd Bnai Zion Medical Center, Haifa, Israel; eee Cedars-Sinai Heart Institute Los Angeles, California, USA; fff The Queen Elizabeth Hospital Woodville, South Australia, Australia; ggg Arthritis and Rheumatology of Georgia Atlanta, Georgia, USA; hhh Cincinnati Children’s Hospital Medical Center Cincinnati, Ohio, USA; iii Edith Wolfson Medical Center Holon, Israel; jjj Rambam Health Corporation Haifa, Israel; a Department of Biomedical and Clinical Science, University of Milan, Fatebenefratelli Hospital, Milan, Italy; b Internal Medicine, Fatebenefratelli Hospital, Milan, Italy; c The Heart Failure Institute, Leviev Heart Centre, Sheba Hospital and the School of Medicine, Tel Aviv University, Tel Aviv, Israel; d Center for the Diagnosis and Treatment of Pericardial Diseases, Section of Cardiovascular Imaging, Department of Cardiovascular Medicine, Heart, Vascular, and Thoracic Institute, Cleveland Clinic, Cleveland, Ohio, USA; e Division of Rheumatology, IRCCS Ospedale Pediatrico Bambino Gesù, Rome, Italy; f Shaare Zedek Medical Center, Jerusalem, Israel; g The Cardiology Unit, University of Vermont Medical Center, Burlington, Vermont, USA; h The Minneapolis Heart Institute, Abbott Northwestern Hospital, Minneapolis, Minnesota, USA; i Division of Cardiovascular Ultrasound, Department of Cardiovascular Medicine, Mayo Clinic, Rochester, Minnesota, USA; j Chaim Sheba Medical Center, Clinical Research Unit, Leviev Heart Center, Ramat Gan, Israel; k Kiniksa Pharmaceuticals, Lexington, Massachusetts, USA; l Department of Medicine (DMED), Cardiothoracic Department, University Hospital “Santa Maria della Misericordia”, ASUFC, Udine, Italy

## Abstract

**Background:**

Rilonacept inhibits the interleukin-1 pathway, and extended treatment in patients with recurrent pericarditis (RP) reduced recurrence risk by 98% in the phase 3 trial, RHAPSODY long-term extension (LTE). Severe acute respiratory syndrome (SARS)-CoV-2 vaccination and/or infection may trigger pericarditis recurrence, and in clinical practice, it is unknown whether to continue rilonacept during SARS-CoV-2 infection. This post-hoc analysis of the RHAPSODY LTE aimed to inform rilonacept management in RP patients vaccinated against SARS-CoV-2 or who contract COVID-19.

**Methods:**

Analysis was conducted from May 2020 to June 2022. The LTE portion of RHAPSODY LTE enabled up to 24 months of additional open-label rilonacept treatment beyond the pivotal study. Rilonacept efficacy data in preventing pericarditis recurrence were assessed, and concomitant SARS-CoV-2 vaccination and COVID-19 adverse event data were evaluated.

**Results:**

No pericarditis recurrences were temporally associated with vaccination. Sixteen COVID-19 cases were reported; 10 in 30 unvaccinated or partially vaccinated patients (33%) vs 6 of 44 fully vaccinated patients (14%; *P* = 0.04). Twelve of 16 patients (75%) were receiving rilonacept at the time of infection, and none experienced pericarditis recurrence. One pericarditis recurrence occurred in the peri-COVID-19 period in 1 of 4 patients who had stopped rilonacept treatment > 4.5 months prior. COVID-19 severity was mild in 13 patients, moderate in 2, and severe in 1.

**Conclusions:**

Full vaccination effectively reduced COVID-19 events in patients treated with rilonacept. Vaccination or COVID-19 during rilonacept treatment did not increase pericarditis recurrence. Continued rilonacept treatment in patients contracting COVID-19 did not worsen disease severity, whereas rilonacept interruption increased pericarditis recurrence, supporting a recommendation for continued rilonacept treatment for RP during vaccination or COVID-19.

**ClinicalTrials.gov identifier:**

NCT03737110

## Résumé

**Contexte:**

Le rilonacept inhibe la voie de l’interleukine-1 et, d’après les résultats de la période de prolongation à long terme de l’essai de phase III RHAPSODY, la poursuite du traitement par cet agent chez les patients atteints de péricardite récidivante a réduit le risque de récidive de 98 %. La vaccination contre le syndrome respiratoire aigu sévère (SRAS)-CoV-2 ou l’infection à ce virus pourrait toutefois déclencher une récidive de la péricardite, et dans la pratique clinique, on ignore s’il vaut mieux poursuivre le traitement par rilonacept pendant l’infection à SRAS-CoV-2. Cette analyse post-hoc de la période de prolongation à long terme de l’essai RHAPSODY vise à orienter la gestion du rilonacept chez les patients atteints de péricardite récidivante qui sont vaccinés contre le SRAS-CoV-2 ou qui contractent la COVID-19.

**Méthodologie:**

L’analyse a été effectuée de mai 2020 à juin 2022. La période de prolongation à long terme de l’essai RHAPSODY a permis d’accumuler des données en mode ouvert pendant une période allant jusqu’à 24 mois au-delà de l’étude pivot. Les données sur l’efficacité du rilonacept en prévention de la récidive de péricardite ont été évaluées, tout comme les données sur la vaccination concomitante contre le SRAS-CoV-2 et les cas de COVID-19.

**Résultats:**

Aucune récidive de la péricardite n’a pu être associée sur le plan temporel avec la vaccination. Au total, 16 cas de COVID-19 ont été signalés, dont 10 chez les patients non vaccinés ou partiellement vaccinés sur 30 (33 %) et 6 chez les patients complètement vaccinés sur 44 (14 %; *p* = 0,04). De ces 16 patients, 12 (75 %) prenaient du rilonacept au moment de l’infection et aucun n’a connu de récidive de la péricardite. Une récidive de la péricardite s’est produite dans la période suivant la COVID-19 chez 1 des 4 patients qui avaient cessé de prendre le rilonacept > 4,5 mois auparavant. La COVID-19 a été légère chez 13 patients, modérée chez 2 patients et sévère chez 1 patient.

**Conclusions:**

La vaccination complète a réduit efficacement les cas de COVID-19 chez les patients traités par le rilonacept. La vaccination ou l’infection à SRAS-CoV-2 pendant le traitement par rilonacept n’a pas augmenté le risque de récidive de la péricardite. La poursuite du traitement par rilonacept chez les patients atteints de COVID-19 n’a pas aggravé la sévérité de la maladie, tandis que l’interruption du traitement a augmenté le risque de récidive de la péricardite, ce qui plaide en faveur de la recommandation de poursuivre le traitement de la péricardite récidivante par le rilonacept pendant la vaccination ou la COVID-19.

Recurrent pericarditis (RP) is a chronic autoinflammatory disease of 3-year median duration requiring prolonged treatment to prevent recurrence.^1^, ^2^, ^3^ The interleukin-1 (IL-1) cytokine family dominates the inflammatory response in RP, and IL-1 pathway inhibition reduces recurrence risk in patients with RP.^4^ Rilonacept reduced pericarditis recurrence risk by 96% (hazard ratio in a Cox proportional-hazards model, 0.04; 95% confidence interval, 0.01-0.18; *P* < 0.0001 by log-rank test) in the phase-3 study **R**ilonacept In**h**ibition of Interleukin-1 **A**lpha and Beta for Recurrent **P**ericarditis: a Pivotal **S**ymptomatology and **O**utcomes Stud**y** (RHAPSODY).^5^ The RHAPSODY long-term extension (LTE) results showed persistent (98%) risk reduction, whereas premature rilonacept cessation increased recurrence risk.^6^

The RHAPSODY LTE took place during the peak of the COVID-19 pandemic. Although COVID-19 was not a primary study outcome, it provided a valuable opportunity for gaining additional on-treatment efficacy and safety insights regarding RP relating to severe acute respiratory syndrome (SARS)-CoV-2 vaccination and COVID-19 infection, refining clinical decision-making regarding rilonacept therapy under these circumstances.

## Methods

### Study design and patients

**Figure 1:**
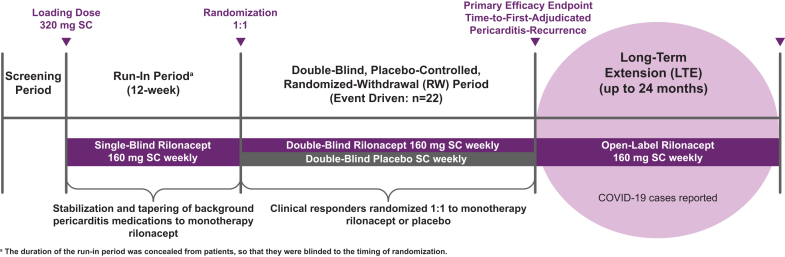
**R**ilonacept In**h**ibition of Interleukin-1 **A**lpha and Beta for Recurrent **P**ericarditis: a Pivotal **S**ymptomatology and **O**utcomes Stud**y** (RHAPSODY) long-term extension (LTE) design—a phase 3, double-blind, placebo-controlled, event-driven, randomized-withdrawal (RW) trial of rilonacept in patients with recurrent pericarditis, which also included a long-term extension phase, allowing up to 24 months of additional open-label rilonacept treatment. SC, subcutaneous.

This was a post-hoc analysis of 74 patients between 12 and 75 years of age participating in the 24-month open-label RHAPSODY LTE (NCT03737110),^5^ conducted from May 2020 to June 2022 (Fig. 1) in 4 countries (n = 45 in the US; n = 29 in Italy, Israel, and Australia). A total of 69 patients completed the RHAPSODY LTE (27 [39.1%] male, 42 [60.9%] female): US sites concluded in April 2021 after approval of rilonacept for use in RP by the US Food and Drug Administration; non-US patients remained until study closure (June 2022).

SARS-CoV-2 vaccines became available in November 2020. The vaccine series type was recorded as concomitant medication. COVID-19 complications were recorded as adverse events and were classified by investigators as mild, moderate, or severe, according to the US Food and Drug Administration adverse-event reporting guidelines.^7^ COVID-19 diagnoses were not adjudicated, and the disease detection method was not reported.

### Outcome measures

Outcome measures included pericarditis recurrences in the setting of SARS-CoV-2 vaccine and COVID-19 cases related to clinical management.

### Statistical analysis

Due to the small sample size and the unplanned pandemic, descriptive summary statistics are provided (n, mean, standard deviation [SD]), using SAS version 9.4 (SAS Institute, Cary, NC).

## Results

### Study population

In total, 74 RHAPSODY patients commenced the LTE (mean [SD] age, 44.2 [15.6] years; 40 women [54.1%] and 34 men [45.9%]; 68 [91.9%] White, 5 [6.8%] Black/African American, and 1 [1.4%] other). Mean RP disease duration at study entry was 2.5 years. Sixty-nine patients completed the RHAPSODY LTE; overall median (maximum) treatment duration was 22 (35) months. In April 2021, after rilonacept approval in the US for treatment of RP and after the US sites concluded, 32 of 45 US patients (71.1%) transitioned to commercial rilonacept after a median (maximum) 18 (27)-month treatment duration. Twenty-five non-US patients remained on rilonacept until study closure (through a 6-week safety follow-up) in June 2022 after a median (maximum) 28 (35)-month treatment duration.

### SARS-CoV-2 vaccination

During the RHAPSODY LTE while on study treatment, 47 of 74 patients (63.5%) received at least 1 SARS-CoV-2 vaccine (no live-attenuated; messenger [m]RNA [n = 34], adenovirus [n = 2], and “unknown” [n = 11]); 44 of 74 (59.5%) were considered fully vaccinated (received a complete vaccine series), and 30 of 74 (40.5%) were partially vaccinated or unvaccinated.

### COVID-19 incidence

No COVID-19 cases were reported during the randomized-withdrawal period (ended May 2020). A listing of prevalent SARS-CoV-2 variants during the conduct of the RHAPSODY LTE is found in Supplemental Table S2.^8^ A COVID-19 adverse event was reported in 16 of 74 patients (21.6%). COVID-19 cases were reported in 10 of 30 unvaccinated or partially vaccinated patients (33%; 13.5% of total enrollment) and in 6 of 44 fully vaccinated patients (14%; 8.1% of total enrollment). Among the 6 COVID-19 cases reported in fully vaccinated patients, 1 occurred about 11 months after, and a second case 14 months after, completion of the vaccine series (Supplemental Table S3).

### COVID-19 severity and rilonacept treatment

COVID-19 severity was mild (n = 13), moderate (n = 2), or severe (n = 1). Twelve infected patients (75%) were receiving rilonacept at the time of COVID-19 diagnosis; 4 (25%) were off-treatment, 2 had previously “suspended” rilonacept for off-treatment observation at the 18-month decision milestone, and 2 were in the 6-week safety follow-up period after having discontinued treatment. Of those patients receiving rilonacept at the time of COVID-19 diagnosis, 10 of 12 (83.3%) continued rilonacept, 1 of 12 (8.3%) interrupted rilonacept for 4 weeks, and 1 of 12 (8.3%) permanently discontinued rilonacept. COVID-19 resolved in all but the 1 patient who permanently discontinued rilonacept; this patient had a prolonged severe COVID-19 course complicated by preexisting comorbidities.

### Pericarditis recurrence outcomes

Among the 12 of 16 patients (75%) receiving rilonacept at the time of infection, no patients (0 of 12) experienced a pericarditis recurrence during or after the COVID-19 adverse event. Of the 2 of 16 patients (12.5%) who had previously “suspended” rilonacept for off-treatment observation at the 18-month decision milestone, 1 investigator-assessed pericarditis recurrence (without C-reactive protein elevation) occurred in the peri-COVID-19 period (18 days after infection), approximately 4.5 months after rilonacept cessation. No pericarditis recurrences were reported in the 2 of 16 patients (12.5%) whose COVID-19 cases occurred after the end of rilonacept treatment visits and during the 6-week safety follow-up period (Table 1). Among the 58 of 74 patients (78%) without a COVID-19 infection, no pericarditis recurrences were temporally associated with SARS-CoV-2 vaccination.

**Table 1:** Severe acute respiratory syndrome (SARS)-CoV-2 infections grouped by vaccination status and whether patients were receiving rilonacept at the time of infection; pericarditis recurrences are also reported

Severity	Patients with COVID infection, n = 16		
	Vaccinated n = 6 (37.5%)	Partially vaccinated or unvaccinated n = 10 (62.5%)	Total n = 16 (100%)
Mild, n = 13	5 of 6 (83.3)	8 of 10 (80.0)	13 of 16 (81.3)
Moderate, n = 2	1 of 6 (16.7)	1 of 10 (10.0)	2 of 16 (12.5)
Severe, n = 1	0	1 of 10 (10.0)	1 of 16 (6.3)
On rilonacept, n = 12	5 of 6	7 of 10	12 of 16 (75.0)
Mild, n = 9	4 of 5 (80.0)	5 of 7 (71.4)	9 of 12 (75.0)
Moderate, n = 2	1 of 5 (20.0)	1 of 7 (14.2)	2 of 12 (16.7)
Severe, n = 1	0	1 of 7 (14.2)	1 of 12 (8.3)
Pericarditis recurrence	0	0	0
Off rilonacept, n = 4	1 of 6	3 of 10	4 of 16 (25.0)
Mild, n = 4	1 of 1 (100)∗	3 of 3 (100)	4 of 4 (100)
Moderate, n = 0	0	0	0
Severe, n = 0	0	0	0
Pericarditis recurrence	0	1 of 3 (33.3)†	1 of 4 (25.0)

Values are n (%).

∗ COVID-19 adverse event was reported during the safety follow-up period, 1 month after the patient had stopped rilonacept treatment.

† One pericarditis recurrence (without C-reactive protein elevation) occurred in the peri-COVID-19 period (18 days after infection), approximately 4.5 months after rilonacept cessation.

## Discussion

RP is a chronic condition, and physicians commonly must decide how to manage treatments during intercurrent events, such as infections or vaccinations, particularly when biologics agents are used. Use of an anti–IL-1 agent during COVID-19 and/or other infections could be useful, and the interruption of the drug might facilitate a recurrence of pericarditis. The RHAPSODY LTE took place during the height of the COVID-19 pandemic, providing the opportunity to study rilonacept use in patients with RP who were vaccinated against SARS-CoV-2 and/or contracted COVID-19.

We observed that rilonacept did not appear to interfere with the protective effect of SARS-CoV-2 vaccination; as expected, patients who were fully vaccinated experienced a lower incidence of COVID-19, compared to patients who were unvaccinated or partially vaccinated in the setting of rilonacept treatment.^9^

An inappropriate and exuberant innate immune response contributes to COVID-19 morbidity, and higher levels of proinflammatory cytokines, including IL-1ß, are associated with more severe disease.^10^ Inhibition of IL-1 signaling reduced mortality and the need for invasive mechanical ventilation among patients hospitalized with COVID-19.^11^ In line with these observations, we found that rilonacept treatment did not appear to impact the safety of patients with COVID-19.

Similar to other viruses, SARS-CoV-2 may retrigger autoinflammatory processes that contribute to pericarditis recurrence. Among patients participating in the RHAPSODY LTE, premature cessation of rilonacept led to pericarditis recurrences, regardless of COVID-19 or vaccination status.

### Limitations

The descriptive analyses reported here were not prespecified, because of the unpredictable circumstances of the pandemic. The sample size related to vaccination and infection was small (44 patients were fully vaccinated, 30 patients were partially vaccinated or unvaccinated; 16 SARS-CoV-2 infections reported), and the cohort was not randomized. Administration of Paxlovid and the presence of anti-SARS-CoV-2 monoclonal antibodies were not captured in this study. Recurrences were investigator-assessed and were not externally adjudicated.

## Conclusions

**Figure 2:**
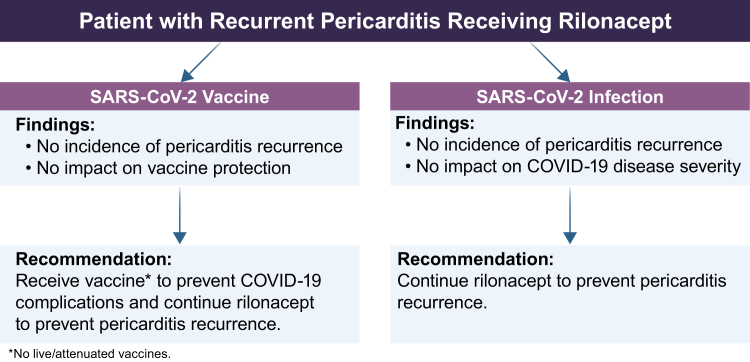
Summary of post-hoc analysis findings and clinical recommendations rubric. Among patients on rilonacept in the **R**ilonacept In**h**ibition of Interleukin-1 **A**lpha and Beta for Recurrent **P**ericarditis: a Pivotal **S**ymptomatology and **O**utcomes Stud**y** (RHAPSODY) long-term extension (LTE) (n = 74), no pericarditis recurrences were associated temporally with severe acute respiratory syndrome (SARS)-CoV-2 vaccination, and no impact on vaccine protection was observed. After SARS-CoV-2 infection, no patients had a pericarditis recurrence, and it had no impact on COVID-19 severity. It is recommended that patients with recurrent pericarditis receiving rilonacept receive a SARS-CoV-2 vaccine and continue rilonacept to prevent pericarditis recurrence.

In this size-limited analysis, rilonacept did not appear to interfere with SARS-CoV-2 vaccine protection. The results suggest that patients with RP being treated with rilonacept should receive a SARS-CoV-2 vaccination series to reduce possible complications from COVID-19. Continued rilonacept treatment during COVID-19 was well tolerated, supporting a recommendation that RP patients should continue rilonacept regardless of COVID-19 status to prevent pericarditis recurrence (Fig. 2). The data presented support the further study of the relationships among RP, vaccination, and infection in the setting of anti-IL-1 therapies.

## References

[1] Imazio M. (2014). Idiopathic recurrent pericarditis as an immune-mediated disease: current insights into pathogenesis and emerging treatment options. Expert Rev Clin Immunol.

[2] Klein A., Cremer P., Kontzias A. (2021). US database study of clinical burden and unmet need in recurrent pericarditis. J Am Heart Assoc.

[3] Lin D., Laliberte F., Majeski C. (2021). Disease and economic burden associated with recurrent pericarditis in a privately insured United States population. Adv Ther.

[4] Del Pinto R., Ferri C. (2021). Recurrent pericarditis is less scary: the new therapeutic solutions. Eur Heart J Suppl.

[5] Klein A.L., Imazio M., Cremer P. (2021). Phase 3 study of IL-1 trap rilonacept in recurrent pericarditis. N Engl J Med.

[6] Imazio M., Klein A.L., Brucato A. (2024). Sustained Pericarditis Recurrence Risk Reduction With Long-Term Rilonacept. J Am Heart Assoc.

[7] US Food & Drug Administration FDA Adverse Event Reporting System (FAERS) public dashboard. https://www.fda.gov/drugs/questions-and-answers-fdas-adverse-event-reporting-system-faers/fda-adverse-event-reporting-system-faers-public-dashboard.

[8] Institute of Social and Preventive Medicine University of Bern, Switzerland, and SID Swiss Institute of Bioinformatics. CoVariants. https://covariants.org/.

[9] Atagunduz P., Keser G., Soy M. (2021). Interleukin-1 inhibitors and vaccination including COVID-19 in inflammatory rheumatic diseases: a nonsystematic review. Front Immunol.

[10] Vora S.M., Lieberman J., Wu H. (2021). Inflammasome activation at the crux of severe COVID-19. Nat Rev Immunol.

[11] Makaremi S., Asgarzadeh A., Kianfar H. (2022). The role of IL-1 family of cytokines and receptors in pathogenesis of COVID-19. Inflamm Res.

