# Supplementary material for: Absence of Pericarditis Recurrence in Rilonacept-Treated Patients With COVID-19 and SARS-CoV-2 Vaccination: Results From the RHAPSODY Long-term Extension
Source: CJC Open. 2024 Mar 4;6(6):805–10. doi: 10.1016/j.cjco.2024.02.002 (PMC11250856; doi:10.1016/j.cjco.2024.02.002)
Supplement: Supplemental Material [file mmc1.docx]

SUPPLEMENTARY MATERIAL

| **Investigator** | **Affiliation** | **Location** |
| --- | --- | --- |
| Antonio Abbate | University of Virginia | Charlottesville, Virginia, USA |
| Wael Abo-Auda | CardioVoyage | McKinney, Texas, USA |
| Asif Akhtar | BI Research Center | Houston, Texas, USA |
| Michael Arad | Chaim Sheba Medical Center | Ramat Gan, Israel |
| Shaul Atar | Galilee Medical Center | Nahariya, Israel |
| Bipul Baibhav | Rochester General Hospital | Rochester, New York, USA |
| Karan Bhalla | Orion Medical | Pasadena, Texas, USA |
| Antonio Brucato | ASST Fatebenefratelli Sacco - Ospedale Fatebenefratelli e Oftalmico | Milan, Italy |
| Sean Collins | Vanderbilt University Medical Center | Nashville, Tennessee, USA |
| David Colquhoun | Core Research Group | Milton, Queensland, Australia |
| Paul Cremer | Cleveland Clinic | Cleveland, Ohio, USA |
| David Cross | HeartCare Partners Clinical Research Unit | Milton, Queensland, Australia |
| Girish Dwivedi | Fiona Stanley Hospital | Murdoch, Western Australia, Australia |
| Alon Eisen | Rabin Medical Center | Petach Tikva, Israel |
| Nahum Freedberg | HaEmek Medical Center | Afula, Israel |
| Shmuel Fuchs | Assaf Harofe Medical Center | Tzrifin, Israel |
| Eliyazar Gaddam | Loretto Hospital | Chicago, Illinois, USA |
| Marco Gattorno | Istituto G Gaslini Ospedale Pediatrico IRCCS | Genova, Italy |
| Eli Gelfand | Beth Israel Deaconess Medical Center | Boston, Massachusetts, USA |
| Paul Grena | Cardiology Consultants of Philadelphia | Yardley, Pennsylvania, USA |
| Majdi Halabi | Ziv Medical Center | Zefat, Israel |
| David Harris | University of Cincinnati | Cincinnati, Ohio, USA |
| Massimo Imazio | Azienda Ospedaliero Città della Salute e della Scienza di Torino | Turin, Italy |
| Antonella Insalaco | Ospedale Pediatrico Bambino Gesù | Rome, Italy |
| Amin Karim | Angiocardiac Care of Texas PA | Houston, Texas, USA |
| Allan Klein | Cleveland Clinic | Cleveland, Ohio, USA |
| Kirk Knowlton | Intermountain Healthcare | Murray, Utah, USA |
| Apostolos Kontzias | Stony Brook University School of Medicine | Stony Brook, New York, USA |
| Robert Kornberg | Icahn School of Medicine at Mount Sinai | New York, New York, USA |
| Faisal Latif | Oklahoma City VA Medical Center – National Association of Veterans’ Research and Education Foundations (NAVREF) | Oklahoma City, Oklahoma, USA |
| David Leibowitz | Hadassah University Hospital Mount Scopus | Jerusalem, Israel |
| Martin LeWinter | University of Vermont Medical Center | Burlington, Vermont, USA |
| David Lin | Minneapolis Heart Institute Foundation | Minneapolis, Minnesota, USA |
| Pey Wen Lou | GenesisCare – Cardiology Research | Doncaster, East Victoria, Australia |
| S. Allen Luis | Mayo Clinic | Rochester, Minnesota, USA |
| Stephen Nicholls | Monash Health, Monash Medical Centre | Clayton, Victoria, Australia |
| John Petersen | Swedish Medical Center | Seattle, Washington, USA |
| Michael Portman | Seattle Children’s Hospital | Seattle, Washington, USA |
| Philip Roberts-Thomson | Royal Hobart Hospital | Hobart, Tasmania, Australia |
| Elad Schiff | Bnai Zion Medical Center | Haifa, Israel |
| Robert Siegel | Cedars-Sinai Heart Institute | Los Angeles, California, USA |
| Michael Stokes | The Queen Elizabeth Hospital | Woodville, South Australia, Australia |
| Paul Sutej | Arthritis and Rheumatology of Georgia | Atlanta, Georgia, USA |
| Samuel Wittekind | Cincinnati Children’s Hospital Medical Center | Cincinnati, Ohio, USA |
| Valentin Witzling | Edith Wolfson Medical Center | Holon, Israel |
| Robert Zukermann | Rambam Health Corporation | Haifa, Israel |

**Supplemental Table S1.** RHAPSODY Investigators.

|  | **Variant** | | | |
| --- | --- | --- | --- | --- |
| **Country** | **641G** | **B.1.1.17** | **B.1.617.2** | **B.1.1.529** |
| Israel | Dec 2020 - Feb 2021 | Mar 2021 - Jun 2021 | Jul 2021 - Dec 2021 | Jan 2022 - Jun 2022 |
| Italy | Jun 2020 | Jul 2020 - Jun 2021 | Jul 2021 - Nov 2021 | Dec 2021 - Jun 2022 |
| United States | Jun 2020 - Mar 2021 | Apr 2021 - May 2021 | Jun 2021 - Dec 2021 | Jan 2022 - Jun 2022 |

**Supplemental Table S2.** SARS-CoV-2 variants by country from June 2020 to June 2022.

| **COVID-19 AE Case** | **Time Between Last Booster Inoculation & COVID-19 AE (months)** | **Severity of Disease** |
| --- | --- | --- |
| Subject #1 | 2.5 | Mild |
| Subject #2 | 3.5 | Mild |
| Subject #3 | 5 | Moderate |
| Subject #4 | 6 | Mild |
| Subject #5 | 11 | Mild |
| Subject #6^a^ | 14 | Mild |

Abbreviations: AE, adverse event.

**Supplemental Table S3.** Patients who had received full-course SARS-CoV-2 vaccination and developed COVID-19. ^a^ COVID-19 AE was reported during the safety follow-up period, 1 month after the patient had stopped rilonacept treatment.
